# Identification of two insecticide resistance markers in Ethiopian *Anopheles stephensi* mosquitoes using a multiplex amplicon sequencing assay

**DOI:** 10.1038/s41598-023-32336-7

**Published:** 2023-04-05

**Authors:** Holly Acford-Palmer, Jody E. Phelan, Fitsum G. Tadesse, Mojca Kristan, Emma Collins, Anton Spadar, Thomas Walker, Teun Bousema, Louisa A. Messenger, Taane G. Clark, Susana Campino

**Affiliations:** 1 Faculty of Infectious and Tropical Diseases, London School of Hygiene and Tropical Medicine, London, UK; 2 Malaria and NTD Directorate, Armauer Hansen Research Institute, ALERT Hospital Compound, P.O. Box 1005, Addis Ababa, Ethiopia; 3 Department of Medical Microbiology, Radboud University Nijmegen Medical Centre, Nijmegen, The Netherlands; 4 Environmental and Occupational Health, School of Public Health, University of Nevada, Las Vegas, Las Vegas, USA; 5 Faculty of Epidemiology and Population Health, London School of Hygiene and Tropical Medicine, London, UK

**Keywords:** Computational biology and bioinformatics, Ecology, Genetics, Genomics, Haplotypes, Population genetics, Sequencing

## Abstract

Since its first detection in 2012 in Djibouti, *Anopheles stephensi* has invaded and established in the Horn of Africa, and more recently Nigeria. The expansion of this vector poses a significant threat to malaria control and elimination efforts. Integrated vector management is the primary strategy used to interrupt disease transmission; however, growing insecticide resistance is threatening to reverse gains in global malaria control. We present a next-generation amplicon-sequencing approach, for high-throughput monitoring of insecticide resistance genes (*ace1,* *GSTe2,* *vgsc* and *rdl*), species identification and characterization of genetic diversity (*its2* and *cox1*) in *An. stephensi*. Ninety-five *An. stephensi* mosquitoes, collected in Ethiopia, were screened, identifying 104 SNPs, including the knock-down mutation L958F (L1014F in *Musca domestica*), and for the first time in this vector species, the A296S substitution (A301S in *Drosophila melanogaster*) in the *rdl* locus. Two other amino acid substitutions (*ace1-*N177D, *GSTe2-*V189L) were also identified but have not been previously implicated in insecticide resistance. Genetic diversity in the mitochondrial *cox1* gene revealed shared haplotypes between Ethiopian *An. stephensi* with samples from Pakistan, Sudan, and Djibouti. Overall, we present a reliable, cost-effective strategy using amplicon-sequencing to monitor known insecticide resistance mutations, with the potential to identify new genetic variants, to assist in the high-throughput surveillance of insecticide resistance in *An. stephensi* populations.

## Introduction

The first confirmed finding of the Asian mosquito, *Anopheles stephensi,* in Africa was reported in Djibouti City in 2012^1^, and it has now spread throughout the Horn of Africa into Ethiopia, the Republic of Sudan, Somalia, and recently Nigeria (2020) and Yemen^2–7^. *Anopheles stephensi* is a highly competent vector for both *Plasmodium falciparum* and *P. vivax* malaria parasites. A primary malaria vector in South Asian countries such as India and Pakistan, *An. stephensi* also present in the Middle East^1^. The spread of this vector into Africa has sparked concerns for the control and elimination of malaria, particularly since its first report in Djibouti malaria cases drastically increased from only 1,684 cases in 2013 to 73,535 in 2020^3,8–10^. Whilst not confirmed, this drastic increase is potentially a result of this vector’s ability to occupy different ecological niches compared to other mosquito species in the region. The primary malaria vector in the Horn of Africa, *An. arabiensis*, transmits malaria in rural areas or extensive areas of urban agriculture ^10–12^. However, *An. stephensi* is a proficient vector in urban areas, being able to use artificial water sources such as water tanks and polluted water sources for larval habitats^13^. As the continent of Africa becomes increasingly urbanised, the spread of *An. stephensi* is predicted to put 126 million Africans, with little to no acquired immunity, at risk of malaria without immediate control^12^.

Over the last two decades, interventions to control mosquito vectors have greatly contributed to the decline in malaria morbidity and mortality. Long lasting insecticidal nets (LLINs) and indoor residual spraying (IRS) have been successfully used to control African malaria vectors such as *An. funestus* and *An. gambiae* sensu lato complex species. However, *An. stephensi* exhibits different feeding (outdoor, evening biting) and resting behaviours (animal shelters are common dwellings) rendering methods such as IRS less effective^12,14^. In addition, the effectiveness of insecticides is threatened by increasing resistance in *Anopheles* vectors, which has been reported in almost all African countries^15,16^.

The main mechanisms associated with insecticide resistance are target site insensitivity and metabolic resistance^17^. Several substitutions in the *voltage-gated sodium channel* (*vgsc*) gene, have been associated with resistance to pyrethroids and dichlorodiphenyltrichloroethane (DDT) (knock down resistance; *kdr*)^18,19^. Mutations and duplications in the *acetylcholinesterase 1* (*ace1*) gene have been associated with resistance to both carbamates and organophosphates^20,21^, whereas mutations in the *GABA* receptor (dieldrin-*rdl* locus) have been associated with resistance to phenylpyrazoles and organochlorides, particularly dieldrin^22–24^. GABA receptors are also believed to be a target of pyrethroids^25,26^. Metabolic resistance resulting from enhanced detoxification of insecticides has been reported as one of the causes of resistance to insecticides from diverse classes and are usually associated with the over-expression of detoxification enzymes^27^. Other mechanisms of insecticide resistance identified include mosquito microbiome components and cuticle alterations^27–30^. Resistance to several insecticides of all major classes has been reported in *An. stephensi* in India, Sri Lanka, many countries of the WHO Eastern Mediterranean Region and also in Ethiopia^20,31–35^. Besides the use of susceptibility bioassays and biochemical techniques to characterize insecticide resistance, the occurrence of the *vgsc*-V1014 *kdr* mutation has also been investigated in *An. stephensi* populations^20,31–35^. In Ethiopia, the L1014F mutation was reported at low frequencies. In a cohort of permethrin-resistant *An. stephensi* the *kdr* mutation was not detected, this suggests other molecular mechanisms maybe involved with pyrethroid susceptibility in this vector. Therefore, additional candidate genes need to be explored to better understand the wider landscape of insecticide-associated mutations in *An. stephensi* populations.

The use of whole genome sequencing (WGS) gives more insight into the genomic landscape of organisms, however limitations such as high cost and low DNA quantities make it an unsuitable method for high-throughput surveillance. The application of multiplex amplicon sequencing with next generation sequencing (NGS) technologies provides a good, high throughput alternative to screen target regions of interest in large datasets, in a cost-efficient manner. Target amplicon sequencing (Amp-seq) is based on the sequencing of pools of PCR amplicons of interest and has been applied in malaria surveillance to characterise *P. falciparum*^36^ and *An. gambiae s.l.* populations^37,38^. Here we designed and validated a targeted Amp-seq assay, combined with dual indexing barcodes and Illumina sequencing, for profiling *An. stephensi* across *vgsc*, *rdl*, *gste2* and *ace1* loci related to insecticide resistance. Further, we included amplicons in the assay for species identification and phylogenetic analysis, specifically the internal transcribed spacer (*its2)* and the mitochondrial gene (*cytochrome c oxidase subunit 1*, *cox1)*. Using this approach, it is possible to pool multiple samples across several loci, distinguishing individual samples based on the unique index barcodes, increasing efficiency, and decreasing costs. Amp-seq can be used to screen for known and novel mutations, which when used in combination with phenotyping assays, can identify genetic variants predictive of resistance to interventions/insecticides. The assay is easy to implement and can be applied to many samples at low cost, achieved through PCR multiplexing and dual barcoding. It is a promising tool to confirm species, support the surveillance of insecticide resistance in *An. stephensi* and inform vector control strategies targeting this invasive species in the African continent.

## Results

### Detection of SNPs in genes associated with insecticide resistance

The amplicon data generated from 95 *An. stephensi* sourced from Ethiopia, identified a total of 104 high quality SNPs and 20 indels (Table 1, Supplementary Table 1). The average coverage per amplicon was high (range: 437.54 to 14,483.47 reads) (Table 1). Most of the SNPs identified were synonymous, not leading to any changes in the protein sequence. Only four SNPs were found to result in a missense mutation, with two previously described to be associated with insecticide resistance (Supplementary Table 2). The *kdr* mutation L958F (L1014F in *M. domestica*) in the 2nd domain of the *vgsc* locus, was detected in 13 Ethiopian samples (13.8%). The other known SNP was a A296S substitution (A301S in *D. melanogaster*) in the *GABA*-gated chloride channel (*rdl* locus), which has been previously reported in *Anopheles* species but not *An. stephensi*^39^. In our study, A296S was detected in 21 (22.1%) Ethiopian samples. Two putatively novel missense variants were identified in our study. First, a SNP in the *ace1* gene (N177D) found in 19 (57.8%) Ethiopian samples and in the insecticide susceptible colony populations (n = 2). Second, a SNP in the *GSTe2* gene (V189L) was detected at low frequency (n = 3). Sanger sequencing confirmed that these SNPs were true polymorphisms and not sequencing artifacts.

**Table 1:** Average amplicon coverage, and number of genetic variants detected.

Amplicon	Average coverage	Number of SNPs/INDELs		
		All	Ethiopia	Colony
ACE1_I	13,624.74	15	12	6
ACE1_II	614.67	14	14	11
CO1	14,483.47	6	6	2
GSTe2	2394.67	16	13	12
ITS2	2231.35	2	2	0
Rdl1	1499.71	20	19	7
Rdl2	1241.53	27	25	14
VGSCI	1242.09	3	2	1
VGSCII	437.54	21	16	12
VGSCIII	750.55	6	4	6
VGSCIV	1841.83	12	11	3

### Genetic diversity of An. stephensi populations from Asia and Africa

**Figure 1:**
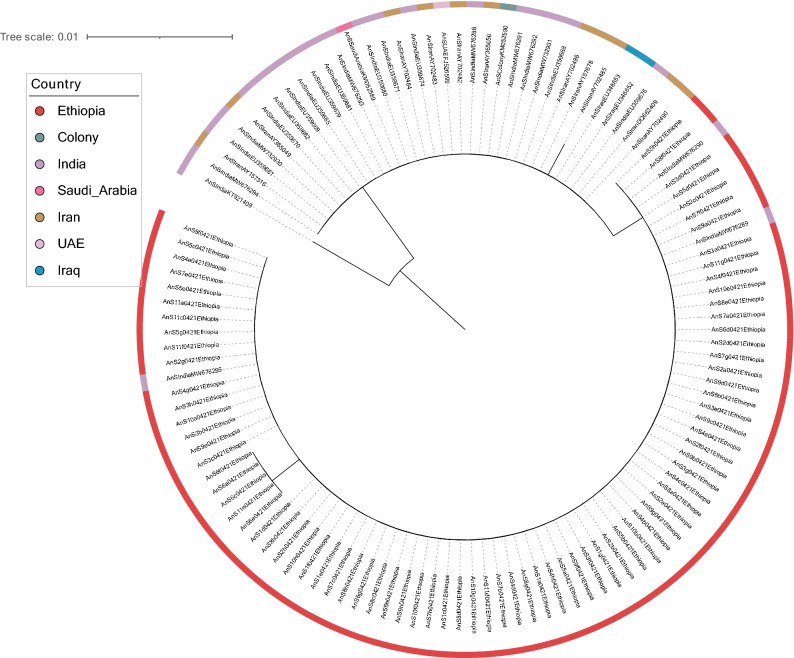
Neighbourhood joining tree constructed using ITS2 gene sequences generated from this study, alongside publicly available sequence data. The tree was built using the maximum-likelihood method assuming GTR model of nucleotide substitution, with the gamma model of heterogeneity rate. This tree was generated using RAxML, and visualised with iTOL^65,66^.

**Figure 2:**
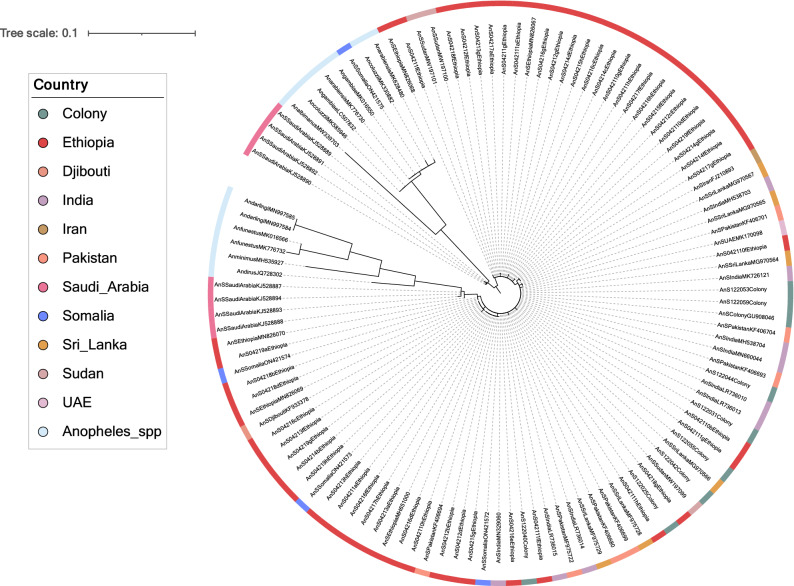
Neighbourhood joining tree constructed using *cox1* gene sequences generated from this study, alongside publicly available sequence data from *An. stephensi* and a variety of other *Anopheles spp*. This includes *An. albimanus, An. arabiensis, An. coluzzi, An. darlingi, An. dirus, An. funestus,* and *An. gambiae*. The tree was built using the maximum-likelihood method assuming GTR model of nucleotide substitution, with the gamma model of heterogeneity rate. This tree was generated using RAxML, and visualised with iTOL^65,66^.

Amplicon sequences of the ribosomal DNA internal transcribed spacers *its2* and mitochondrial *cox1* gene were analysed alongside publicly available data. Across 81 Ethiopian samples, 38 publicly available sequences and 1 colony (SDA500 colony specimen, descended from a Pakistani isolate), only two SNPs were identified in *its2*, one present only in 6% of Ethiopian samples, and the other present in all Ethiopian samples and a few samples from India. A few singletons were also found. Phylogenetic analyses revealed a clade that includes all Ethiopian samples and three samples from India (Fig. 1), while the samples from other geographic regions clustered together with no specific geographic cluster observed, reflecting the limited genetic diversity at this locus, also supported by the low nucleotide diversity (π = 0.00169). For the *cox1* gene, 49 Ethiopian samples, 41 publicly available sequences and 1 colony sample were analysed. Two SNPs were identified in Ethiopia (22%; 38%), and some singleton SNPs were also observed. For the phylogenetic analysis, additional *cox1* sequences from other *Anopheles* species, (*An. albimanus*, *An. arabiensis*, *An. coluzzii*, *An. darlingi*, *An. dirus*, *An. funestus*, *An. gambiae*, *An. minimus*), were also included. The phylogenetic analyses revealed a clade that separated the non-*An. stephensi* samples, including a subclade with 3 samples from the *An. gambiae* complex. For the remaining *An. stephensi*, due to the limited genetic diversity at this locus, supported by the low nucleotide diversity (π = 0.00521), there were no specific geographic clusters observed (Fig. 2). The nucleotide diversity was in accordance with data previously reported for this species at this locus^40^.

**Figure 3:**
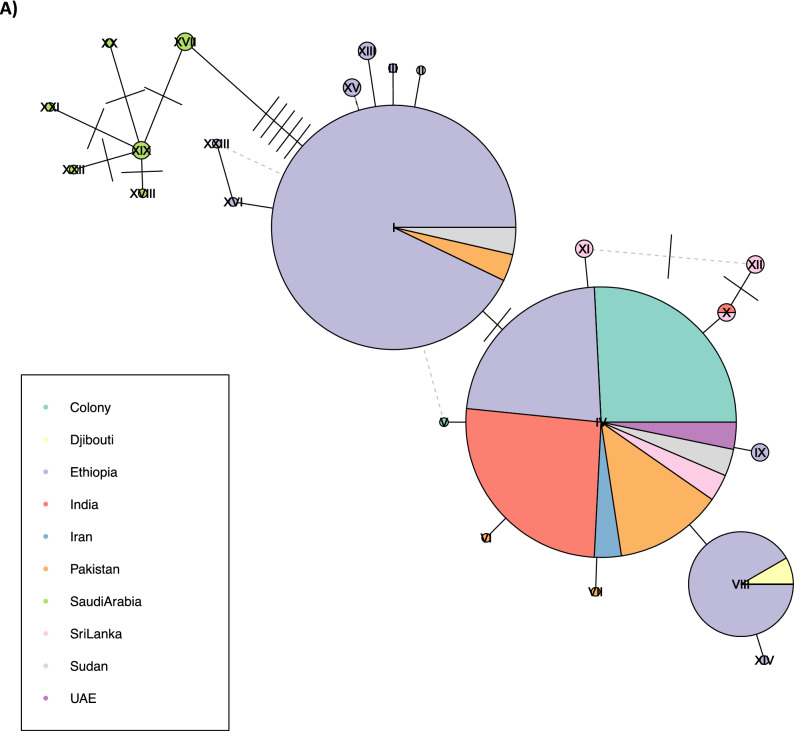

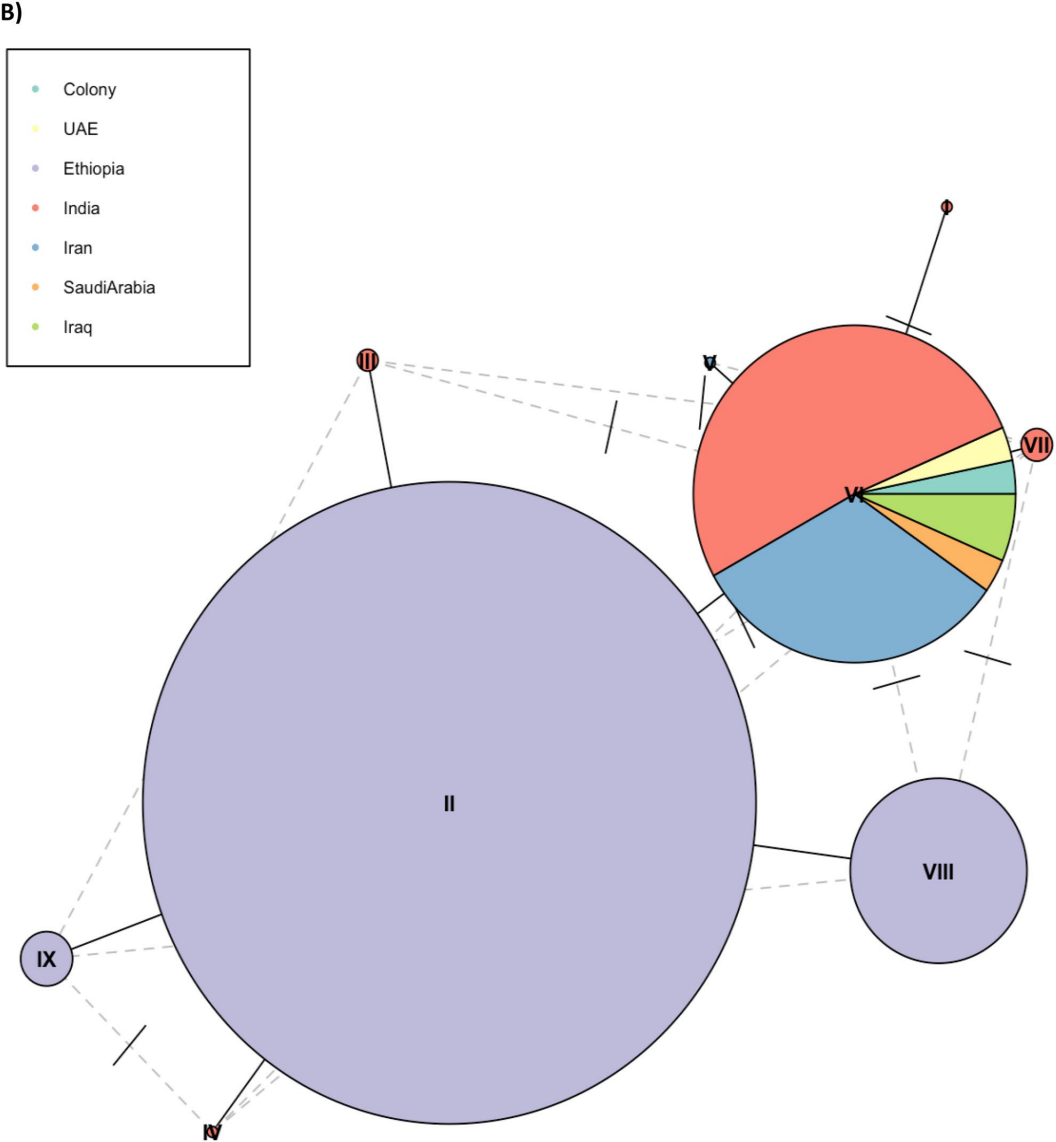
Haplotype or minimal-spanning network constructed using *cox-1* sequences (A) and *its2* sequences (B) generated in this study alongside publicly available data. Each node represents a haplotype, with each segment in the node representing a country, proportionally sized to the number of samples present in the segment and node. The number of nucleotides differences between haplotypes is represented by the number of ticks between nodes.

Haplotype networks were constructed to analyse and visualize the relationships among the DNA sequences (Fig. 3). For *its2*, nine haplotypes were identified, with > 72% of Ethiopian samples sharing a single haplotype. The samples from other geographic regions also shared a core haplotype. More haplotypes were identified for *cox1* (n = 24), with a core haplotype present in almost all samples from the different countries, except Saudi Arabia that did not share any haplotypes with other populations. A single sample from India also shared a less frequent haplotype with a Sri Lankan specimen. For the samples from Ethiopia, besides the core haplotype, other haplotypes were also observed, particularly one shared with samples from Pakistan and Sudan, and a less frequent haplotype shared with samples from Djibouti.

## Discussion

The introduction of *An. stephensi* mosquitos into the Horn of Africa is a significant threat to malaria control. Vector control strategies have been successful in reducing malaria transmission^41,42^ and surveillance of insecticide resistance is essential to inform malaria control programmes. To assist surveillance activities, we evaluate an Amp-seq assay to screen for known and novel mutations associated with a range of insecticides. In the *An. stephensi* specimens screened, the *kdr* mutation L958F (L1014F in *M. domestica;* L1014F *kdr-west*) was present at a frequency of 10.2%. The L1014F *kdr-west* mutation was first detected in *An. gambiae* in West Africa. It was reported at a low frequency (5.7%) in Ethiopian *An. stephensi* specimens collected in 2018 from other regions (Bati, Degehabur, Dire Dawa, Gewane, and Semera)^34^. The same study also screened samples from Awash Sebat Kilo, the region where this studies samples were collected, but did not detect this mutation. However, a year later, we have detected the L1014F *kdr-west* mutation. This may be a result of this studies larger sample size (n = 95), compared to n = 8. This mutation has also been confirmed in populations of *An. stephensi* in the Middle East and South Asia, with both *kdr-west* (L1014F) and *kdr-east* (L1014S) being described^20,31,32,43–45^.

In this study we also identified the A296S mutations in the GABA gene (*rdl*). This mutation has not been reported previously in *An. stephensi*, likely because this locus has not been screened in this mosquito species. Dieldrin, alongside other cyclodienes that target the GABA receptor, are no longer officially used due to environmental toxicity and the potential impact on human health. However, the A296S mutation may confer resistance to other insecticides, such as fipronil; and broflanilide. These are newly discovered GABA-gated chloride channel allosteric modulators under evaluation as an IRS tool^46,47^, so it remains important to identify and document its presence. We targeted two other mutations in the *rdl* gene (V327I and T345S), which are seen in tandem with the A296S SNP^39,48,49^. However, we found no evidence of these non-synonymous SNPs in the specimens analysed. These secondary SNPs have been described in both Asian and African *Anopheles* mosquito populations, but always in the presence of the A296S mutation^39,48,49^. No other commonly reported mutations, such as the *ace1* G119S, were identified in the specimens analysed. However, two new mutations were detected in the Ethiopian samples: N177D in the *ace1* gene, and V189L in the *GSTe2* gene. Both were also present in insecticide susceptible colony samples, suggesting these mutations are not involved in insecticide resistance.

The genetic analysis using data from *its2* and *cox1* genes revealed a low level of genetic diversity, consistent with studies of native *An. stephensi* populations in Sri Lanka and India, where few variants and minimal genetic differentiation in these loci was observed^13,50–52^. For *its2*, the Ethiopian samples cluster together and separate from the *An. stephensi* from other countries. The Ethiopian samples were all collected in the same village, likely explaining the clustering pattern observed. More haplotypes were observed for the *cox1* gene, with a core haplotype shared by all samples, except those sourced from Saudi Arabia. Ethiopian samples shared haplotypes with samples of Pakistan origin, as previously reported^53^, but also shared haplotypes with samples from neighbouring countries (Sudan, Djibouti), supporting evidence of the spread of this vector across the Horn of Africa. It is possible that this vector is already present in other regions in Africa, as recently reported in Nigeria and Kenya (year 2020)^54,55^. Strengthening the surveillance activities of this species, through large-scale genetic characterisation of this vector, is urgently needed to rapidly implement targeted control strategies.

Overall, the cost-effective and high throughput Amp-seq approach presented here, can be implemented by vector control programs to screen *An. stephensi* mosquitoes for both known and putative novel insecticide resistance mutations. A limitation of our approach is that it only targets loci that are established mechanisms of insecticide resistance. However, it is easy to add other targets to the Amp-seq method as new loci are detected. This approach can be used to complement diagnostic bioassays, by providing genotyping data alongside phenotypic information, to detect new genetic variants underlying insecticide resistance. Our approach is highly flexible, with the easy addition of genomic regions of interest to the panel, for example, to include loci related to metabolic resistance or that can target pathogens such as malaria parasites, thereby enhancing surveillance activities.

## Conclusion

This study validated the use of an amplicon sequencing panel to perform molecular monitoring of *An. stephensi* insecticide resistance and explore genetic diversity. The identification of two known SNPs associated with insecticide resistance, alongside other non-synonymous SNPs not previously described in any mosquitoes highlights the potential for this technology to find existing and novel mutations that could affect insecticide response. Here we confirm the presence of the *vgsc*-L1014F *kdr* mutation and, for the first time in this species, the A296S *rdl* mutation. We were also able to investigate the genetic structure of *An. stephensi* and haplotype sharing between this Ethiopian population and neighbour countries. The extension of the approach to other loci can further assist with supporting control strategies of vector-borne diseases and reducing their global burden.

## Materials and methods

### Mosquito collection and identification

*An. stephensi* mosquitoes were obtained from the LSHTM colony (Sind Kasur strain, from Pakistan), and field samples were collected in Awash Sebat Kilo, Ethiopia. Samples collected in Ethiopia were caught between April and September 2019, in one of three ways: CDC mini light traps, aspiration from cattle shelters, and human landing collection. The mosquitoes were first identified morphologically as *An. stephensi*, before undergoing a qPCR targeting the *ITS2* and *cox1* genes for molecular confirmation using previously described methods^9^.

### DNA extraction

Individual mosquitoes were suspended in 1X PBS and lysed mechanically for 30 s or until all body parts were no longer visible using a Tissue Ruptor II (Qiagen, Hilden, Germany). The DNA was then extracted using Qiagen DNAeasy Blood and Tissue kits, according to manufacturer’s instructions. DNA for each sample was quantified using Qubit 2.0 fluorimeter HS DNA kit (ThermoFisher, Waltham, MA, USA) and stored at − 20 °C.

### Primer design for Amp-seq

Sequences were downloaded from VectorBase (https://vectorbase.org/vectorbase/app). The primers were designed using Primer3^56^ and aimed to amplify an approximately 500 bp region, which contained previously described SNPs associated with target site resistance in *Anopheles* and other vectors (Supplementary Table 3). Primers were designed to bind to exonic regions where possible. Primers were designed to target 7 genomic regions in SNPs previously associated with insecticide resistance: 4 different domains of the voltage-gated sodium channel (*vgsc* DI-IV), one partial sequence of the gene *acetylcholinesterase 1* (*ace1*), one sequence in the resistance to dieldrin gamma-amino butyric acid receptor (*rdl*), and another in the *gste2* gene (Supplementary Table 4). Two genomic regions were targeted for species identification and/or phylogenetic analyses: the nuclear ribosomal internal transcribed spacer 2 (*ITS2*) and the mitochondrially encoded cytochrome c oxidase I (c*ox1*), (Supplementary Table 3). The primer sequences were concatenated with unique 5’ tag barcode (6 bp long) to discriminate individual samples and enable multiplexing, along with sequences complementary to Illumina adapters for sequencing as described previously^38^. Prior to PCR amplification, each sample was assigned one unique barcode in each forward and reverse primer to be used for amplification of all loci. To identify primers suitable for multiplexing, ThermoFisher Scientific Multiple Primer Analyser was used with sensitivity set to one to identify potential primer dimerization events.

### Amplicon generation and next generation sequencing

Amplicons were generated using NEB Q5 Hot-start polymerase (NEB, New England Biolabs, UK) in 25ul final reactions. Final primer concentration averaged 0.5um for all assays, and 1 μl (around 2 ng/μl) of sample extract was used. The cycling conditions were as follows: hot start polymerase activation for 3 min at 95 °C, then 30 cycles of 95 °C for 10 s, 58 °C for 30 s and 72 °C for 45 s, followed by a final elongation step of 72 °C for 5 min. After gene-specific multiplex PCR reactions, amplicons were visualized in a 1% agarose gel to check for band size and intensity. Each PCR multiplex from each mosquito sample were pooled together, and then pooled with other mosquitoes that had different 5’tag barcodes to reach a maximum of 200 amplicons per pool. Pools were purified with using Kapa beads (Roche) as per the manufacturer’s instructions in a 0.7:1 bead to sample volume ratio to remove excess primers and PCR reagents. The concentration of each pool was then measured using the Qubit 2.0 fluorimeter HS DNA kit (ThermoFisher, Waltham, MA, USA). The final pool containing 200 amplicons was then used as the template in the indexing PCR (second PCR step), which was performed to introduce the Illumina adaptors and barcode. This 2nd PCR step was performed as part of the Illumina-based Amplicon-EZ service (Genewiz, UK), followed by sequenced using Illumina MiSeq, on a configuration of paired end 2 × 250 bp. A minimum of 50,000 reads were obtained per pool (250 reads per amplicon in a pool of 200 amplicons) using the Genewiz service (< US$ 0.5 per amplicon).

### Amplicon bioinformatic analysis

Raw sequences were de-multiplexed based on the barcode combination assigned to each sample using an in-house python script (https://github.com/LSHTMPathogenSeqLab/amplicon-seq). The resulting raw sequence data was then analysed using an in-house pipeline, where raw sequence data for each sample was trimmed using Trimmomatic software, mapped to the reference sequence (UCI_ANSTEP_V1.0) using bwa-mem software, and reads clipped using Samclip package with a maximum clip length of 50^57–59^. Sample fastq files are available at European Nucleotide Archive website (Project ID: PRJEB57331,

Accession numbers: ERR10484018—ERR10484147).

Multiple variant callers were utilised to maximise the number of mutations identified, and as an additional quality control procedure. SNPs and small indels were called using freebayes (v1.3.5, –haplotype-length -1) and GATK HaplotypeCaller (v4.1.4.1, default parameters) software tools^60,61^. The variants were then filtered (DP > 30) using bcftools software to ensure only high-quality variants were included. High quality SNPs were identified using filters that included a minimum phred quality of 30 per called base, minimum depth of 30 reads, and minimum alternate allele depth of 10. Finally, only SNPs that were present in > 1 sample, and present across two independent pools were retained. Variant annotation was carried out using the SnpEff tool, combined with a database based on the UCI_V1 reference genome^62,63^. The percentage of alternative allele to total depth coverage was used to classify genotypes to homozygous reference (< 25% alternate allele reads), homozygous alternate (> 75% alternate allele reads) or heterozygous (25–75% alternate allele), as described previously^38^.

### Phylogenetic analysis

For the *ITS2* and *cox1* amplicons, following SNP calling, all bam files from samples with > 50-fold coverage for these genes were converted to fasta files using an in-house pipeline (https://github.com/LSHTMPathogenSeqLab/fastq2matrix). The FASTA files were then aligned using MAFFT for each gene. The alignments also included 37 *ITS2 An. stephensi* nucleotide sequences public available (accession numbers: India (n = 22, EU359661, EU359662, EU359665, EU359668-EU359671, U359674, EU359676, U359679, EU359680, EU359681, KT921409, MW676288-MW676295, MW732930), Iran (n = 11, AY157316, AY157678, AY365049, AY365050, AY702482, AY702483, AY702484, AY702485, AY702486, AY702490, DQ662409, Iran (n = 2, EU346653, EU346652), Saudi Arabia (KM052589), UAE (FJ526599)). For *cox1*, alignments also included 40 nucleotide sequences public available (accession numbers: Saudi Arabia (n = 8, KJ528887- KJ528894), Djibouti n = 1(MK170098), Ethiopia (n = 5,MH651000, MN826067-MN826070), India (n = 9, LR736010, LR736013- LR736015, MH538703, MH538704, MK726121, MN329060, MN660044) Iran (FJ210893), Pakistan (n = 7 , KF406680, KF406693, KF406694, KF406699, KF406704, KF406704), Sri Lanka (n = 6, MF975728, MF975729, MG970564-MG970567), Sudan (n = 3, MW197099-MW197101), UAE (MK170098)). For COI other Anopheles species were included (*An. albimanus* (MW339703), *An_arabiensis*_MK628480, *An. coluzzii* (MK330882), *An. darlingi* MN997585, *An. dirus* JQ728302, *An. funestus* MK776732, *An. gambiae*_LC507832, *An. minimus* MH535927). The resulting alignment was then viewed and trimmed on Aliview software^64^. Following this, a phylogenetic tree was constructed using RAxML software^65^. RAxML uses a maximum-likelihood approach, and the GTRGAMMA option applied which assumes GTR model of nucleotide substitution, with the gamma model of heterogeneity rate. A bootstrap value of 500 replicates was used to construct the tree. Trees were then visualised in the iTOL tool^66^.

### Haplotype networks

The aligned FASTA files for ITS2 and *cox1* genes, were used to construct haplotype networks using the Pegas package in R^67^. The same package was also used to calculate nucleotide diversity, haplotype diversity and Tajima’s D statistic.

## Supplementary Information

Supplementary Tables.

## Data Availability

All raw sequence data is listed in the European Nucleotide Archive (Project ID: PRJEB57331, Accession numbers: ERR10484018—ERR10484147).
