# Supplementary material for: Identification of two insecticide resistance markers in Ethiopian Anopheles stephensi mosquitoes using a multiplex amplicon sequencing assay
Source: Sci Rep. 2023 Apr 5;13:5612. doi: 10.1038/s41598-023-32336-7 (PMC10076309; doi:10.1038/s41598-023-32336-7)
Supplement: Supplementary file 1 — Supplementary Tables. [file 41598_2023_32336_MOESM1_ESM.docx]

**Supplementary** **Table 1**. Variants Identified in each population

**Supplementary** **Table 2**. Locations of the four missense SNPs identified in all samples

| **Sample** | **Sample Number** | **Number of SNPs** | **No. of Missense SNPs** | **Number of INDELs** |
| --- | --- | --- | --- | --- |
| All | 127 | 115 | 4 | 27 |
| Ethiopia | 95 | 104 | 4 | 20 |
| Colony | 33 | 63 | 1 | 11 |

| **Gene** | **Chromosome** | **Position** | **Amino Acid** |
| --- | --- | --- | --- |
| *ace1* | NC_050202.1 | 60913884 | N177D |
| *gaba (rdl)* | NC_050203.1 | 8353055 | A296S |
| *vgsc* | NC_050203.1 | 42817709 | L958F |
| *gste2* | NC_050203.1 | 70580373 | V189L |

**Supplementary** **Table 3.** Locations of insecticide resistance associated SNPs with targeted amplicons

| **Target Gene** | **Amplicon** | **Target SNP** | **Reference Species** | **Location in *An. stephensi*** | **Described in *An. stephensi*** | **First described in** |
| --- | --- | --- | --- | --- | --- | --- |
| *ace1* | ACE1_I | G119S | *T.californica* | G276S | N | *D. melanogaster^1^* |
|  | ACE1_II | N485I |  | N639I | N | *Anopheles funestus^2^* |
| *gste2* | GSTe2 | L119F | *An. funestus* | L119F | N | *An. funestus^3^* |
| *vgsc* | VGSCI | V410L | *M.domestica* | V371L | N | *Aedes aegypti^4^* |
|  | VGSCII | L1014F |  | L958F | Y ^5,6^ | *An.gambiae^7^* |
|  | VGSCIII | F1552C |  | F1569C | N | *Ae.aegypti^8^* |
|  | VGSCIV | D1794Y |  | D1783Y | N | *Ae. aegypti^9^,* |
| *gaba*  *(rdl)* | RDL1 | A302S | *D. melanogaster* | A296S | N | *D. melanogaster^10^* |
|  | RDL2 | V327I |  | V327I | N | *An. funestus^11^* |
|  | RDL2 | T345S/M |  | T345S | N | *Anopheles sinensis^12^* |

**Supplementary Table 4.** Amplicon Primer sequences with target SNPs

| **Associated with** | **Target Gene** | **Amplicon** | **Accession ID** | **Target SNP** | **Exon Span** | **Forward primer** | **Reverse Primer** | **Product Size (bp)** |
| --- | --- | --- | --- | --- | --- | --- | --- | --- |
| Insecticide  Resistance | *vgsc* | VGSCI | ASTEI08161 | V410L | 9 | TTTCTCCAGCACCAAACATT | TGCTCCAAAAATGAACAAAAA | 499 |
|  |  | VGSCII |  | L1014F | 26 | GATTGTGTTCCGTGTGCTGT | GGTTGGTAGCGGTAAGGTGA | 498 |
|  |  | VGSCIII |  | F1152C | 36 | TCTTCGGATCGTTCTTCACC | AGATTCCACGGCTCGATAAA | 496 |
|  |  | VGSCIV |  | D1794Y | 39 | AAGAGTGGTCTGGACGATGTG | GTAGTCGTCGTCGGTCAAGC | 523 |
|  | *ace1* | ACE1_I | ASTE007197 | G119S | 3 | AGGTTCCGTCATCCTCGAC | GGAAACAGCACCAGCACTCT | 483 |
|  |  | ACE1_II |  | N485I | 5 | GTGGGCGATTACCATTTCAC | GGTGCTGCCACTTGTAGGTT | 452 |
|  | *gste2* | GSTe2 | ASTE016034 | L119F | 3 | ATCATTACCGAGAGCCATGC | CACCGTTAGCCTCCTCGTAG | 490 |
|  | *gaba* | rdl1 | ASTE016089 | A296S/V327I | 7 | AGTTTGTACGTTCGATGGGTTA | AGTGGCAGAAAGTGGTGTCC | 498 |
|  |  | rdl2 |  | T345S | 8 | GTCGTTCAGCGCACCACT | GTGAGCTTTCGGGTCGTG | 399 |
| Phylogeny | *cox1* | COI | AF116835.1 | n/a | n/a | AATTAGGACACCCAGGAGCA | GCTCCAGCTAATACAGGTAATGA | 490 |
|  | *its2* | ITS2 | AY157678.1 | n/a | n/a | TCGATGAAGACGCAGCTAAA | GCAACTGGATGCGAGGAA | 492 |
